# Supplementary material for: Introgression of chromosomal segments conferring early heading date from wheat diploid progenitor, Aegilops tauschii Coss., into Japanese elite wheat cultivars
Source: PLoS One. 2020 Jan 27;15(1):e0228397. doi: 10.1371/journal.pone.0228397 (PMC6984701; doi:10.1371/journal.pone.0228397)
Supplement: S1 Table — (PDF) [file pone.0228397.s001.pdf]

S1 Table. Primer list and PCR conditions for the PCR analyses of wheat major genes

Gene	Primer	Sequence (5'-3')	Annealing temp (℃)	Elongation time (sec)	Cycle number
VRN-A1	Ex1/C/F	GTTCTCCACCGAGTCATGGT	55.6	40	30
	Intr1/A/R3	AAGTAAGACAACACGAATGTGAGA			
vrn-A1	Intr1/C/F	GCACTCCTAACCCACTAACC	56	70	30
	Intr1/AB/R	TCATCCATCATCAAGGCAAA			
VRN-B1	Intr1/B/F	CAAGTGGAACGGTTAGGACA	58	50	30
	Intr1/B/R3	CTCATGCCAAAAATTGAAGATGA			
vrn-B1	Intr1/B/F	CAAGTGGAACGGTTAGGACA	56.4	80	30
	Intr1/B/R4	CAAATGAAAAGGAATGAGAGCA			
VRN-D1	Intr1/D/F	GTTGTCTGCCTCATCAAATCC	61	110	30
	Intr1/D/R3	GGTCACTGGTGGTCTGTGC			
vrn-D1	Intr1/D/F	GTTGTCTGCCTCATCAAATCC	61	70	30
	Intr1/D/R4	AAATGAAAAGGAACGAGAGCG			
VRN-A1a	VRN-A1 hexa L	CGGGCAAACGGAATCTACCA	68	60	30
	VRN-A1 hexa R	TGGGGCATCGTGTGGCT			
Ppd-A1a	TaPpd-A1prodelF1	CGTACTCCCTCCGTTTCTTT	55	30	35
	TaPpd-A1prodelR2	GTTGGGGTCGTTTGGTGGTG			
Ppd-A1b	TaPpd-A1prodelF1	CGTACTCCCTCCGTTTCTTT	55	30	35
	TaPpd-A1prodelR3	AATTTACGGGGACCAAATACC			
Ppd-B1	TaPpd-B1proF1	ACACTAGGGCTGGTCGAAGA	64	60	35
	TaPpd-B1int1R1	CCGAGCCAGTGCAAATTAAC			
Ppd-D1a	TaPpd-D1_F1	ACGCCTCCCACTACACTG	55	30	35
	TaPpd-D1_R2	CACTGGTGGTAGCTGAGATT			
Ppd-D1b	TaPpd-D1_F1	ACGCCTCCCACTACACTG	55	30	35
	TaPpd-D1_R1	TGTTGGTTCAAACAGAGAGC			
Pina-D1	Pina_F1	GGTCAGATTCAGTACACGGAACATCACATA	58	30	30
	Pina_aR1	CTTTGCTTGCCCGATCACGCTG			
	Pina_bR1	TTCAAGGTCAATGGGCGCCACTATAA			
Pinb-D1	Pinb_F2	CAACCCATCTATTCATCTCCACCACC	58	30	30
	Pinb_R2	TCACCAGTAATAGCCACTAGGGAACT			
	Pinb_bR1	GGATGCTCACAGCCGCT			
Glu-D1	GDx_F	TTTGGGGAATACCTGCACTACTAAAAAGGT	63	30	30
	GDx5_F	AAAAGGTATTACCCAAGTGTAACTTGTCCG			
	GDx_R	AATTGTCCTGGCTGCAGCTGCGA			
Glu-B3	B3g-17	CACAACAGCAACCATGTTCAC	60	50	30
	B3g-02	GGAATACCTTGCATGGGTTC			
