# Supplementary material for: Introgression of chromosomal segments conferring early heading date from wheat diploid progenitor, Aegilops tauschii Coss., into Japanese elite wheat cultivars
Source: PLoS One. 2020 Jan 27;15(1):e0228397. doi: 10.1371/journal.pone.0228397 (PMC6984701; doi:10.1371/journal.pone.0228397)
Supplement: Attachment — Submitted filename: ResponseToReviewer-Takumi et al-Dec19.docx [file pone.0228397.s002.docx]

PONE-D-19-31756

**Introgression of chromosomal segments conferring early heading date from wheat diploid progenitor, *Aegilops tauschii* Coss., into Japanese elite wheat cultivars**

Dear Editors and reviewers,

Thank you very much for the reviewers’ kind comments and suggestions to our manuscript.

We examined all of the comments, and revised our manuscript according to the comments as followed.

Improved sentences and words were marked by red color in text and tables.

In addition, we improved the format of our manuscript to fit the PLoS ONE’s style requirements.

To Reviewer #1,

Thank you very much for your kind review and polite comments.

1. Please clarify the primers information used in this study.

**<response>** We made a supplementary table for the primer list and PCR conditions.

2. Please unify the font size in Tables and all the manuscript.

**<response>** We improve the font size in Tables to 12 point.

3. Please unify the decimal reservation.

**<response>** We improved Table 2 to unify the decimal reservation.

To Reviewer #2,

Thank you very much for your polite comments.

1. The lines used in the study were derived from only two or three times backcross with elite wheat cultivars. It's very difficult to distinguish the genes related to the early heading traits of the derived lines were from Ae. tauschii or the recombination or gene interactions between elite wheat and durum wheat, and it is proved by the fact that no shared sub-genomic D regions detected in those lines from the same cross in this study. More times backcross (>5 times) should be applied to determine D chromosomes regions related to early heading.

**<response>** The objective of this research is not production of near-isogenic lines for wheat minor QTLs. As described in Introduction, this research is trial to establishment of early heading lines selected based on their phenotype and to determine the commonly shared chromosomal segments from their parental synthetic wheat hexaploid. We determined the parental synthetic wheat-derived chromosomal regions commonly shared in the selected lines. Based on the results, we discussed relationship between the chromosomal regions and presence of the QTLs for heading date in Discussion by referring to previously published reports. Surely, further researches should be required to clarify whether the identified chromosomal regions in fact control the heading date. Therefore, we added a following sentence in the fifth paragraph of Discussion; ‘Further studies to produce near isogenic lines of each minor QTL and to narrow down the introgression regions should be required to identify the causal genes for these QTLs’.

2. By the way, early heading is controlled by QTLs, the chance using major gene controlling the heading date leading to overlap of the harvesting time should be few.

**<response>** We agree with the reviewer’s opinion. So, we think that the phenotype-based selection is important to produce early heading lines with some or several days-earliness especially via minor QTLs. Now, we can analyze the whole-genome genotypes using the next generation to identify easily the introgression regions from the parental wild wheat relatives. We think that this point is important in this study as described in Discussion.

We believe that the revised manuscript is now suitable for publication. We look forward to hearing from you at your earliest convenience.

Yours sincerely,

Shigeo Takumi

(Corresponding author)
